# Atypical presentation of eczematiform dermatophytid secondary to *Trichophyton rubrum* onychomycosis: a case report and literature review

**DOI:** 10.11604/pamj.2024.49.122.45946

**Published:** 2024-12-16

**Authors:** Rachid Douge, Mourad Belaouni, Fouzia Merhari, Manal El Yadari, Mohammed Er-Rami

**Affiliations:** 1 Department of Medical Biology, University Hospital Center of Tangier, Tangier, Morocco,; 2 Abdelmalek Essaâdi University, Faculty of Medicine and Pharmacy, Tangier, Morocco,; 3 Mohammed V University, Faculty of Medicine and Pharmacy, Rabat, Morocco,; 4 Sidi Mohamed Ben Abdellah University, Faculty of Medicine and Pharmacy, Fez, Morocco

**Keywords:** Dermatophytid, onychomycosis, *Trichophyton rubrum*, case report

## Abstract

Dermatophytids are inflammatory dermatological manifestations, due to immuno-allergic reactions and hypersensitivity to the antigens of a fungal pathogen. We report a rare case of dermatophytid secondary to Trichophyton rubrum onychomycosis, which is unusual due to the causative agent and also given the location and appearance of the lesions. This is a 54-year-old male, with no particular pathological history. He presented with recurrent eczematous and papular skin lesions on both lower limbs for 2 years, associated with neglected toenail onychomycosis, which preceded the skin lesions by 18 months. Biological investigations were unremarkable. Mycological samples were taken from skin lesions and nails of which the mycological examination revealed Trichophyton rubrum onychomycosis for nail samples, while that of the skin rash samples was negative. In view of this picture, which was suggestive of dermatophytid, the patient was prescribed terbinafine with a strong local corticosteroid, and the evolution under treatment was marked by progressive healing of skin lesions. This clinical case highlights an unusual pathology in which the diagnosis is not always obvious and often misknowned by many practitioners.

## Introduction

Dermatophytids are defined as aseptic inflammatory dermatological manifestations, having diverse and polymorphic expression, due to immuno-allergic reactions and hypersensitivity to the antigens of a fungal pathogen [1]. These reactions may be localized or generalized, depending on the host's immune response and may occur spontaneously or after initiation of antifungal treatment [2,3].

It is an uncommon pathology, for which the literature data are relatively limited. We report here a rare case of dermatophytid, which is unusual due to the causative agent and also given the location and appearance of the lesions. Through the analysis of this case report and of a literature review on this subject, we highlight the epidemiological, clinical, biological and therapeutic aspects of this pathology, which is largely misknowned and underestimated by many practitioners.

## Patient and observation

**Patient information:** this is a 54-year-old male, immunocompetent, with no particular pathological history, notably of diabetes, psoriasis or other autoimmune disorders. He had not taken any medication in the days preceding the appearance of the lesions, nor of traveling to an area endemic for cutaneous leishmaniasis.

**Figure 1:**
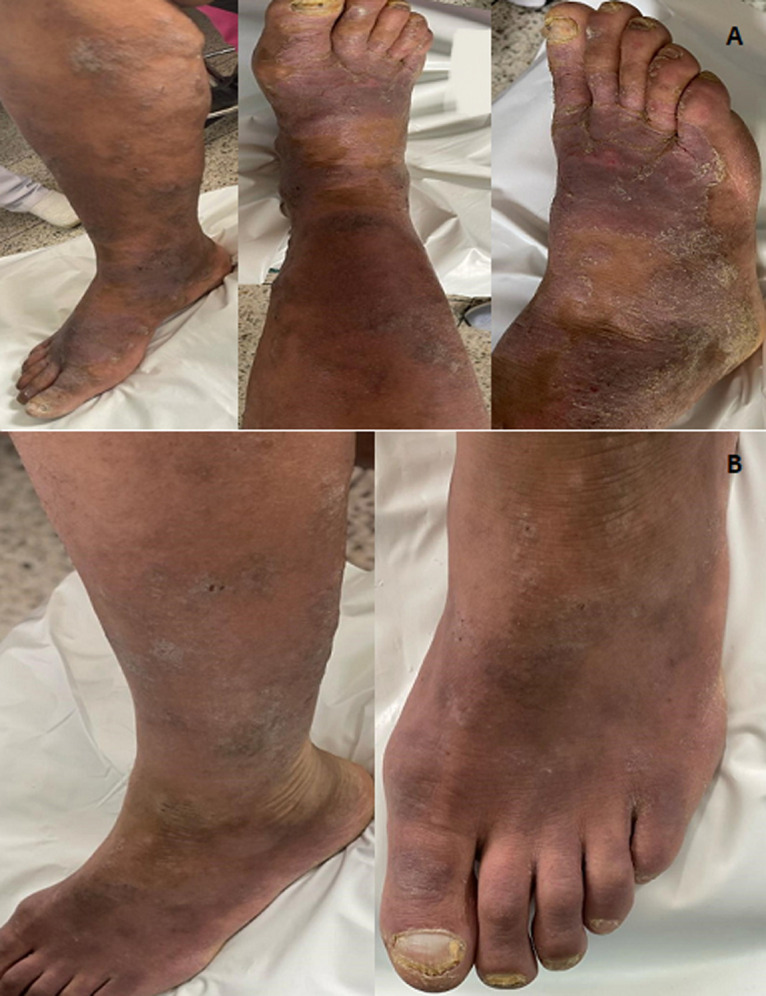
A) extensive eczematous and lichenified lesions on the right leg and foot; B) erythematosquamous and papular lesions of the left leg and foot with onychomycosis

**Clinical findings:** the clinical examination found a patient in a good general condition, apyretic, presenting eczematous, lichenified, erythematosquamous and papular skin eruptions, associated with scratching lesions. These dermatoses are bilateral, localized mainly on the leg and foot, and are more pronounced and more extensive on the right lower limb (Figure 1). Examination also revealed a toenail onychomycosis, which was distolateral, characterized by hyperkeratosis, onycholysis and xanthonychia (Figure 1).

**Timeline of current episode:** the history of the disease dates back 2 years, with the sudden appearance on both lower limbs of skin lesions that were initially erythemato-vesicular, highly pruritic and later evolved into desquamation. This skin rash has preceded by neglected toenail onychomycosis by 18 months. The recurrent nature of the lesions, which relapsed following several courses of treatment based on oral and topical corticosteroids with oral antibiotics and antihistamines drugs, motivated the consultation of the patient in our hospital.

**Figure 2:**
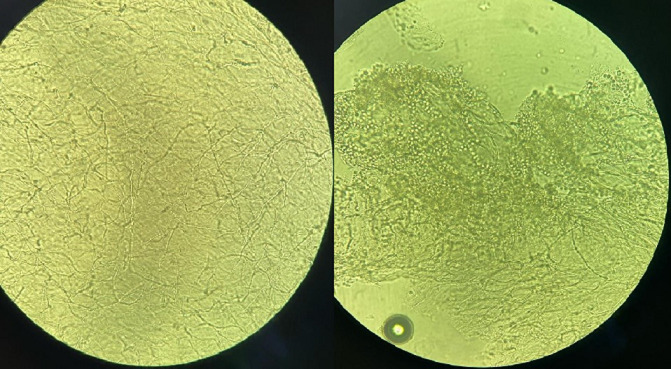
microscopic examination (x40) of nail samples after clearing with potassium hydroxide (KOH 10%), showing the presence of mycelial filaments and spores

**Figure 3:**
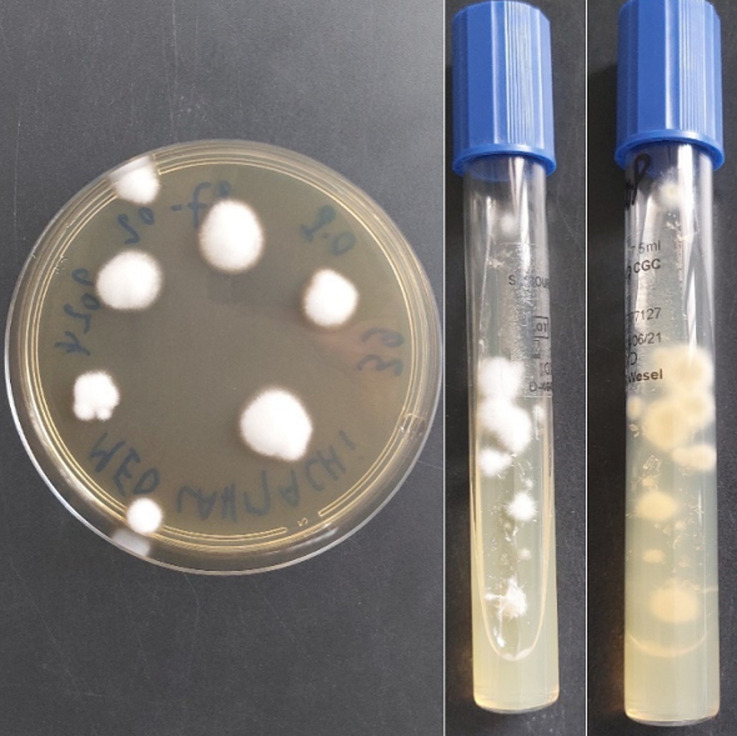
macroscopic examination of positive cultures from nail samples on Sabouraud chloramphenicol and Sabouraud chloramphenicol cycloheximide (Actidione®) media

**Figure 4:**
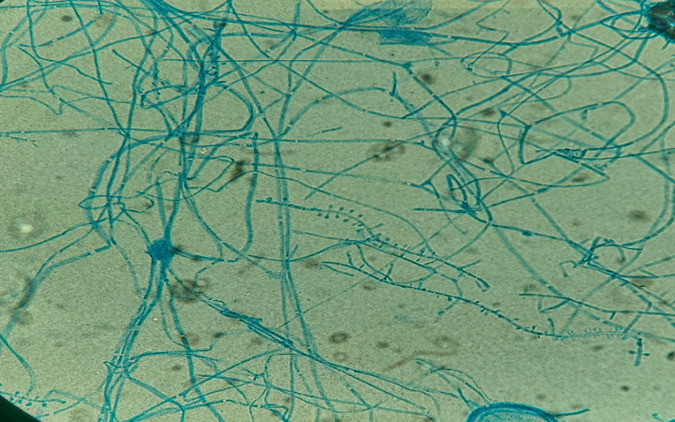
microscopic examination (x100) of positive cultures with lactophenol blue in favor of *Trichophyton rubrum*

**Diagnostic assessment:** biological investigations carried out were unremarkable apart from a moderate increase in C-reactive protein (CRP) analysis (52 mg/l). Samples of skin lesions and nails were taken using sterile scalpel blades after disinfection with physiological water at the different sampling sites. Direct examination between slide and coverslip, after clearing with a potassium hydroxide (10%) preparation, showed the presence of mycelial filaments and spores in the nail samples (Figure 2), whereas it was negative in the skin lesions samples. Direct examination, after scraping the skin lesions with slide spreading, drying and May-Grünwald Giemsa (MGG) staining, did not reveal the presence of fumagoid cells or leishmanial bodies. The samples taken were inoculated in Sabouraud-chloramphenicol and Sabouraud-chloramphenicol-cycloheximide (Actidione®) culture media, then incubated at 28°C and the cultures were examined every 48 hours. On the 6^th^ day of culture, a growth was observed in the culture media from the nail samples, whereas the cultures from the skin samples were sterile after 4 weeks of incubation. Macroscopic examination of positive cultures showed pleated colonies with a downy appearance, whitish on the front and yellowish-brown on the back (Figure 3). Microscopic examination of the cultures with lactophenol blue revealed fine septate filaments with a few branches and some piriform microconidia arranged in an acladium pattern, while macroconidia were not visualized (Figure 4). The diagnosis of *Trichophyton rubrum* onychomycosis was retained for nail disease.

**Diagnosis:** given these anamnestic, clinical and biological data, the diagnosis of dermatophytid was strongly suspected.

**Therapeutic interventions:** in face to the suspicion of dermatophytid secondary to *Trichophyton rubrum* onychomycosis, a treatment based on oral terbinafine 250mg and strong topical corticosteroid (Betamethasone 0.05%) was prescribed for this patient, for 6 months then renewed for a further 3 months.

**Figure 5:**
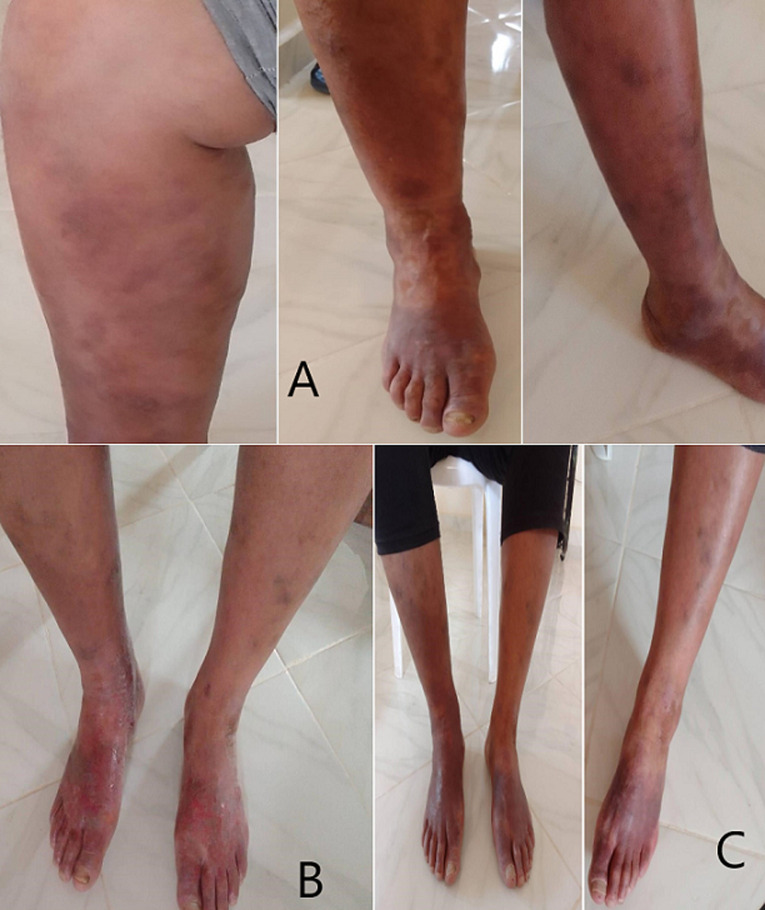
A) post-therapeutic evolution of lesions at 2 months; B) 4 months; and C) 6 months of treatment

**Follow-up and outcome of interventions:** the evolution under treatment was marked by a clear improvement with a progressive regression of the skin lesions, which began first in the areas furthest from the fungal focus, then in a descending manner, with a complete scarring of the rash on both legs at 4 months of treatment, and a healing that is not yet fully complete but nearly, of the feet and of the onychomycosis at 6 months of treatment (Figure 5).

**Patient perspective:** the patient was satisfied to have finally understood the cause of his recurring illness and to see the progressive healing of its lesions. He felt better now in his daily activities, and promised to be still cooperative and to continue his treatment to ensure complete healing of all lesions.

**Informed consent:** it was obtained from the patient for the use of his pictures and clinical data for publication and academic research.

## Discussion

*Trichophyton rubrum* is a dermatophytic, anthropophilic fungus, which has been isolated in more than 80% of dermatophytosis cases at the laboratory in humans [4]. It has also been considered a major fungal agent of the feet, and may be responsible for onychomycosis, intertrigo of plantar and of large folds, as well as palmoplantar pachydermia and circinate dermatophytosis, and more rarely incriminated in perifolliculitis, sycosis and scalp ringworm in children [2]. Therefore, this species is more adapted to humans and rarely responsible for an intense local inflammatory process, due to its inhibitory effect on cellular chemotaxis and thanks to its genesis of mycotoxins and cell wall mannans that have immunosuppressive and immunomodulatory activity on lymphocytes at the local area [4]. However, delayed hypersensitivity to trichophytic antigens may develop in some subjects [5].

It has been assumed that the incidence of dermatophytids is largely underestimated, although data on this subject are poor and vary considerably from one publication to another. This may be explained, on the one hand, by the fact that these lesions were long misknown by practitioners and often wrongly attributed to toxidermia, particularly immediately after the start of antifungal treatment, which leads to the release of excess fungal antigens by the systemic route, this sometimes further accentuates the reactive manifestations [4,6], and on the other hand by the superinfection of lesions, which makes diagnosis of this entity even more difficult [7]. To this effect, Dostrovsky *et al*. [8] reported that only 0.2% of patients with scalp ringworm had presented dermatophytid, while in the series by Grappel *et al*. [5] the occurrence of dermatophytid was noted in 4.2% of children with scalp ringworm and in 4.6% of adults with foot dermatophytosis, although Veien *et al*. [2] have noted that these reactions complicated almost 17% of foot dermatophytosis in general and 7% of dermatophytosis due to *Trichophyton rubrum* especially.

According to the literature, the diagnosis of dermatophytid is often based solely on clinical criteria, mainly a proven dermatophyte fungal infection, a skin rash for which the mycological sample is negative (it is located at a distance from the fungal focus in most of cases), a positive trichophytin skin test (although this test is not necessary in a clinical situation strongly suggestive of dermatophytid, and is generally rarely performed in routine practice), and finally, healing of the rash after treatment of the dermatophytosis [2-4,7].

In our case, the diagnosis of dermatophytid was made in the face of an infection well confirmed by direct examination and culture, which were in favor of *Trichophyton rubrum* onychomycosis, as well as the mycological examination of the skin lesions reactive to this infection which was negative, while the trichophytin skin test was not performed, given that we don´t have access to a validated trichophytin antigen and because of the clinical picture which was suggestive, all the more so as the evolution under treatment which was favorable and marked by a notable regression and a progressive healing of the lesions.

This entity is a rarely reported form of dermatophytid, particularly regarding the localization of lesions in both lower extremities. Indeed, the majority of described cases of dermatophytids secondary to dermatophytosis of the feet reported generally vesicular dermatoses, often bilateral and localized in the hands [1,2,4,7], and lesions located on the face, on the trunk and around the ears for dermatophytids secondary to ringworms of the scalp [3]. However, atypical locations of these dematophytids have also been reported including generalized rashes [6,9], as well as the case described by Tanimura *et al*. [10], who reported an atypical presentation of dermatophytid secondary to *Trichophyton rubrum* onychomycosis in a patient presenting with a generalized pruritic rash cockade shape to type of erythema multiforme-like.

The general clinical presentation of dermatophytids is highly variable, depending on the site of primary infection, the pathogen and also the host. However, the main clinical manifestations of these reactions reported in the literature were usually erythemato-vesicular eruptions, morbilliform, eczematiform, psoriasiform, scarlatiniform, urticarial, lichenoid, erysipeloid lesions and sometimes in the form of erythema multiforme or erythema nodosum and to a lesser degree bullous eruption, centrifugal annulare erythema and angioedema-like reaction [3,4,6,9].

The therapeutic management of this clinical entity is not well codified due to the rarity of its cases and its diagnostic difficulty, but according to several publications, it is mainly based on general and/or topical corticosteroid therapy combined with antifungal treatment, and sometimes supplemented by antibiotic therapy if there is a risk of superinfection of the cutaneous lesions [2,4,6].

## Conclusion

Dermatophytid is a reactive pathology, complicating deep or superficial fungal infections that are not or poorly treated, especially dermatophytosis. They are generally benign but sometimes bothersome functionally and aesthetically. Although their diagnosis is clinical, it is not always obvious, and they are frequently confused with other dermatoses, which justifies the need to research and treat any dermatophytic starting point, in particular onychomycosis which is often neglected, given their long-term asymptomatic nature.
